# Secondary Epileptogenesis: Common to See, but Possible to Treat?

**DOI:** 10.3389/fneur.2021.747372

**Published:** 2021-12-06

**Authors:** Yujia Shen, Yiwei Gong, Yeping Ruan, Zhong Chen, Cenglin Xu

**Affiliations:** ^1^Key Laboratory of Neuropharmacology and Translational Medicine of Zhejiang Province, School of Pharmaceutical Science, Zhejiang Chinese Medical University, Hangzhou, China; ^2^Key Laboratory of Medical Neurobiology of National Health Commission and Chinese Academy of Medical Sciences, Institute of Pharmacology and Toxicology, College of Pharmaceutical Sciences, Zhejiang University, Hangzhou, China

**Keywords:** secondary epileptogenesis, mechanisms, pharmacotherapy, neuromodulation, neural circuits

## Abstract

Secondary epileptogenesis is a common phenomenon in epilepsy, characterized by epileptiform discharges from the regions outside the primary focus. It is one of the major reasons for pharmacoresistance and surgical failure. Compared with primary epileptogenesis, the mechanism of secondary epileptogenesis is usually more complex and diverse. In this review, we aim to summarize the characteristics of secondary epileptogenesis from both clinical and laboratory studies in a historical view. Mechanisms of secondary epileptogenesis in molecular, cellular, and circuity levels are further presented. Potential treatments targeting the process are discussed as well. At last, we highlight the importance of circuitry studies, which would further illustrate precise treatments of secondary epileptogenesis in the future.

## Introduction

Epilepsy is one of the most common neurological diseases, affecting nearly 70 million people with ~1% incidence worldwide (1, 2). Patients with epilepsy suffer from unpredictable seizures. Seizures are characterized by synchronous neural firing originating from the seizure focus. However, more than one epileptic focus may emerge in some patients as the disease progresses, defined as secondary epileptogenesis (3). Frank Morrell initially put forward the term secondary epileptogenesis to describe an independent epileptic focus localized in the homotopic area of the primary focus in the contralateral hemisphere (4). The existence of a secondary focus may lead to further pharmacoresistance and failure of surgical intervention. Although the phenomenon of secondary epileptogenesis has been recognized for long, its mechanism is still “tales from the mist.” Hypotheses include excitatory actions of glutamate, depolarized GABA transmission, and long-term alteration of synaptic plasticity. Here, we first summarized the characteristics of secondary epileptogenesis by reviewing both clinical and experimental studies, followed by different aspects of mechanisms. Then available and possible treatments for interfering secondary epileptogenesis in the recent decades were presented. Given the significant advances of experimental approaches, including optogenetics, neuroimaging, and electrophysiology, we suggest understanding secondary epileptogenesis in a circuitry view and proposing open questions for future direction to improve the management of this common and intractable clinical situation.

## Secondary Epileptogenesis

### Early Findings From Animal Experiment

For many years, it has been known that epileptic discharge in one hemisphere may be related to synchronous discharges in the symmetric region of the other hemisphere (5). However, the precise definition of secondary epileptogenesis was not introduced until the 1960s. Morrell confirmed that after forming the primary epileptic focus, an independent epileptic focus could develop in the symmetric regions (which was also defined as the mirror focus) (4). In another early study, researchers proposed that the direct callosal junction between the primary and the mirror focus is indispensable (6). They questioned whether transecting the corpus callosum in the early stage can prevent secondary epileptogenesis. To address this issue, experiments were firstly performed on cats and rabbits. Ethyl chloride spray was delivered to a small section of the pial surface to produce a relatively small epileptogenic lesion. Recording electrodes were implanted into the sprayed area as well as the symmetric region to record electrical activities. After a few hours, the spike activities in the lesion could be observed. However, several days later, the paroxysmal independent discharges could be recorded by the contralateral electrode. And the callosal transection could prevent this process. Thus, it could be concluded that the callosal pathway is probably the critical route for epileptic propagation to the mirror focus in secondary epileptogenesis (7). In 1975, secondary epileptogenesis was further confirmed in the hippocampal kindled cats by Sato. In that study, epileptic electroencephalographic (EEG) activities were found in different regions apart from the mirror focus (8).

As for rodents, in 1962, Dow et al. firstly reported the development of secondary epileptogenesis on rats. Ten to twenty days after the cobalt application, which would produce a chronic discharging focus at the right cerebral cortex, the epileptic activities were observed at the contralateral hemisphere (9). Another study from Levin et al. confirmed that the contralateral focus, which would show recurrent spontaneous seizures, was not rare in an ethyl chloride freezing induced epilepsy model (10). To analyze the secondary epileptogenesis in rats in detail, Engel applied cobalt powder into the posterior brain of the rats to induce spontaneous epileptic seizures. They found that an independent secondary epileptic focus was easy to develop in the cobalt-induced epileptic model. Furthermore, the secondary epileptic focus was usually more excitable than the primary ones in the latter stage (11). A further histological study found that the RNA level was decreased, and ganglioside sialic acid amount was increased in the secondary focus (12). In addition to the chemical convulsant, kainite acid (KA) induced secondary focus by intra-hippocampal and intra-amygdala microinjection into the mice brain (13). Similar results about secondary epileptogenesis were found on other animal species, regardless of the diverse approaches to induce epileptic seizures. As Engel et al. proposed, the subsequent application of pentylenetetrazol could easily induce epileptic discharges in the contralateral secondary focus at dry ice treated rabbit's cortex (14).

It has to be admitted that using chemical convulsants to induce epileptic seizures has a major limitation. The uncertainty of drug diffusion may further influence other extensive regions beyond the injection site. Thus, further validation of secondary epileptogenesis with a more specific primary focus is necessary. Repetitive electrical stimulation in a routine region was commonly used to investigate the progressive increase of behavioral and EEG seizures (15). Secondary epileptogenesis caused by electrical stimulation has been reported in multiple animal species, including frog, caiman, opossum, and monkey (16, 17). All the animals developed projected or evoked epileptic discharge in the homotopic area. Among them, the squirrel monkeys and rhesus monkeys had the fully independent mirror focus in which the epileptic discharges at the contralateral cortex have no correlation in timeline with that of the primary focus. Furthermore, the time courses of secondary epileptogenesis in lower mammals (e.g., rats, cats, and rabbits) seem to be shorter than those of non-human primates (18).

Besides *in vivo* studies, *in vitro*, ictal models are also beneficial for studying secondary epileptogenesis. In 1997, Khalilov et al. firstly established a well-designed three-chamber *in vitro* model by placing the intact hippocampal structures of neonatal male *Wistar* rats in different compartments of a chamber. Those well-isolated chambers separated by latex membranes could be perfused with different solutions separately (19, 20). Using this *in vitro* model, researchers could study the generation of synchronized neuronal activities and investigate the propagation of local epileptic excitability to distant areas. In their study, the contralateral hippocampus developed an independent epileptic focus after KA treatment on the primary focus despite the application of tetrodotoxin on the commissural fibers to block the neural connections reversibly (21). This *in vitro* preparation of intact hippocampi demonstrated the existence of secondary epileptogenesis in the isolated chamber and could be a promising model to study its mechanisms.

Substantial findings obtained from different models have confirmed the existence of secondary epileptogenesis in different epileptic models (Table 1). The most common localization of the secondary focus is the homotopic area contralateral to the primary focus, and the time of formation may be related to the intrinsic epileptic characteristics of the primary focus. However, due to the limitations of experimental techniques, seizures of these early laboratory experiments were mainly triggered by convulsants or electrical stimulations and were characterized by relatively extensive lesion area and vague primary focus (mainly localized in the cortex). Given that only a certain amount of patients had one specific epileptic focus, we suggest that the chronic spontaneous epileptic models may be more appropriate to study the mechanisms and discover potential therapeutic targets for secondary epileptogenesis.

**Table 1:** Secondary epileptogenesis in animal models.

**Years**	**Authors (Ref.)**	**Species**	**Epileptogenic factors**	**Primary focus**	**Secondary focus**
1947	Pacella et al. (5)	Monkey	Hydrous oxides of aluminum	Motor cortex	Contralateral motor cortex
1959	Morrell (4)	Rabbit	Ethyl chloride spray	Right cortex	Left cortex
1960	Morrell (7)	Cat	Ethyl chloride spray	Right cortex	Left cortex
1962	Dow et al. (9)	Rat	Cobalt powder	Right frontal lobe	Left frontal lobe
1967	Levin and McCrimmon (10)	Rat	Ethyl chloride spray	Right somatosensory cortex	Left somatosensory cortex
1968	Engel (11)	Rat	Cobalt powder	Left posterior portion	Right posterior portion
1968	Wilder et al. (16)	Frog, cayman, opossum, monkey	Freeze lesion, penicillin	Left cortex	Right cortex
1970	Engel and Morrell (14)	Rabbit	Slivers of dry ice	Right cortex	Left cortex
1972	Westmoreland et al. (12)	Rat	Cobalt powder	Right somatosensory area	Left somatosensory area
1975	Morrell et al. (17)	Frog	Electrical stimulation	Right hippocampal cortex	Left hippocampal cortex
1975	Sato (8)	Cat	Electrical stimulation	Left hippocampus	Right hippocampus
1978	Schwarcz et al. (22)	Rat	Kainic acid	Hippocampus	Contralateral hippocampus
1980	Ben-Ari et al. (6)	Rat	Kainic acid	Amygdala, caudate-putamen, globus pallidus, bed nucleus of the stria terminalis and septum	Contralateral homotopic area
1983	Jibiki et al. (23)	Rabbit	Electrical stimulation	Right visual cortex	Left visual cortex
1991	Kirkby et al. (24)	Rat	Electrical stimulation	Right hippocampus	Left hippocampus
1993	Beldhuis et al. (25)	Rat	Electrical stimulation	Amygdala	Contralateral amygdala
1993	Hiyoshi et al. (26)	Cat	Electrical stimulation	Right amygdala	Left mygdala
1994	Szente and Boda (27)	Rat	4-aminopyridine	Cortex	Contralateral cortex
1996	Federico and MacVicar (28)	Guinea pig	Electrical stimulation	Lateral entorhinal cortex (*in vitro*)	Contralateral lateral entorhinal cortex (*in vitro*)
1997	Forti et al. (29)	Guinea pig	Bicuculline	Right anterior piriform cortex (*in vitro*)	Left anterior piriform cortex (*in vitro*)
1997	Kudo et al. (30)	Cat	Electrical stimulation	Right motor cortical	Left motor cortical
1997	Mihaly et al. (31)	Rat	4-aminopyridine	Right frontal neocortex	Left frontal neocortex
2000	Barna et al. (32)	Rat	4-aminopyridine	Right somatosensory cortex	Left somatosensory cortex
2003	Gajda et al. (33)	Rat	4-aminopyridine	Somatosensory cortex	Contralateral somatosensory cortex
2003	Khalilov et al. (21)	Rat	Kainic acid	Right hippocampus (*in vitro*)	Left hippocampus (*in vitro*)
2005	Arabadzisz et al. (34)	Mouse	Kainic acid	Right hippocampus	Left hippocampus
2005	Gajda et al. (35)	Rat	4-AP	Right cortex	Left cortex
2008	Mouri et al. (36)	Mouse	Kainic acid	Amygdala	Contralateral amygdala
2009,2011	Nardou et al. (37–39)	Rat	Kainic acid	Right hippocampus (*in vitro*)	Left hippocampus (*in vitro*)
2013	Sobayo and Mogul (40)	Rat	Kainic acid	Hippocampus	Contralateral hippocampus
2017	Ito et al. (41)	Rat	Hyperthermia	Right hippocampus	Left hippocampus
2017	Kuang et al. (42)	Rat	Electrical stimulation	Right amygdala	Left amygdala

### Clinical Evidence of Secondary Epileptogenesis

Interestingly, the secondary epileptogenesis phenomenon was first presented in animal experiments, and clinicians took many years to validate secondary epileptogenesis in patients. The secondary focus, which can generate separate epileptic seizures in patients, was first reported in 1984. Morrell reviewed patients with benign brain tumors in whom the lesion stayed relatively stable over time. However, EEG recordings confirmed that 34% of those patients could develop an independent secondary epileptogenic focus remote from the tumor sites (13, 43).

The phenomenon of secondary epileptogenesis could be further identified in patients with progressive epilepsy. In those patients, determination of the epileptogenic zone has become a considerable challenge. In a study in 1970, EEG parameters, including the distribution of the beta rhythms and the behavior of the bilateral spike, were analyzed after repetitive injections of thiopental sodium in a total of 82 patients, aiming to guide the epilepsy surgery. The simply routine EEG criteria appeared unreliable for those patients who had widespread epileptic lesions (44). Similarly, Gollwitzer et al. reviewed video-EEG recordings from 100 patients with temporal lobe epilepsy (TLE) and found that the bilateral independent interictal epileptiform activities could be detected in 64% of patients. Their findings suggested that seizure foci were localized in both hemispheres (45). Schmidt et al. reported the phenomenon of seizure recurrence after discontinuing anti-seizure drugs (ASDs) in six patients who had undergone epilepsy surgery (most are temporal lobe surgery). This phenomenon could be attributed to the formation of secondary epileptic foci (46). Another evidence of secondary epileptogenesis was that some patients could develop different types of seizures later in the disease course, with an epileptic focus distinct from the primary site. For instance, Morrell reported a patient who developed a new seizure type (automatism followed by head and eye turning to the left) distinct from the habitual seizures with epigastric sensations followed by lip-smacking. (47). These seizures were distinct from the primary ones as a consequence of the formation of other epileptic foci. As mentioned above, the phenomenon of secondary epileptogenesis was reported more often in patients with temporal or frontal lobe epilepsy and rarer in occipital lobe epilepsy. However, Kim et al. reported an exception in occipital lobe epilepsy. The patient had relapses of seizures 10 months after resecting the defined seizure focus located at the left occipital lobe. Further validation confirmed the formation of a secondary focus located in the homotopic area of the right occipital lobe (48).

Clinical evidence on secondary epileptogenesis is still lacking, limited to case reports or series with small sample size. It could occur in different types of epilepsy (Table 2). Systematic and comprehensive prospective cohort studies are still needed to assess the prevalence and incidence of secondary epileptogenesis in different types of epilepsy and identify factors related to this phenomenon.

**Table 2:** Secondary epileptogenesis in patients.

**Years**	**Authors**	**Sample size**	**Conclusion**
1952	Tukel and Jasper (49)	31 patients with parasagittal epileptogenic lesions	Epileptogenic foci in the cortex near the corpus callosum can cause widespread discharges at both hemisphere.
1961	Falconer and Kennedy (43)	7 patients with small focal lesions (glial hamartomas, angiomas, or other neoplasms)	The EEG disclosed there were bilateral, independent spike discharges in both temporal regions.
1961	Rovit et al. (50)	20 patients	Unilateral carotid amobarbital injection at primary epileptogenic lesions can inhibit bilateral discharges.
1970	Lombroso and Erba (44)	82 patients presenting variety of seizures	Patients with widespread brain involvement in seizure activity are unappropriate for surgery.
1985	Morrell (13)	47 patients with cerebral tumor seen as epilepsy	34% of patients had bilateral, independent epileptiform discharge in their EEGs, and more than one seizure type.
1994	Gilmore et al. (51)	22 patients with complex partial seizures and had temporal lobe neoplasms	The mirror focus is not a contraindication to operation even when the preponderance of interictal discharge is contralateral to the tumor.
1997	Eliashiv et al. (52)	60 patients who had standard en bloc anterior temporal lobe resection	The seizure recurrence at sites distant to the lesion may relevant to years of uncontrolled seizures.
1998	Morris et al. (53)	38 patients with intractable epilepsy and ganglioglioma	Despite years of medically resistant seizures, patients with ganglioglioma can still have good surgical outcomes.
2006	Kimiwada et al. (54)	14 children with partial epilepsy involving the temporal lobe	Radiographic results show the recruitment of hippocampal and thalamic in epileptic network.
2008	Surges et al. (55)	14 patients with tonic–clonic seizures of extrahippocampal onset	Repeated extrahippocampal seizures can result in persistent modifications in hippocampal excitability.
2010	Bortolato et al. (56)	One patient with bilateral foci in frontal lobe	The density of GABA_A_/benzodiazepine receptor binding in the mirror focus had a significant increase.
2014	Kim et al. (48)	One patient with intractable occipital lobe epilepsy	Occipital lobe epilepsy can also have the mirror focus.
2017	Gollwitzer et al. (45)	100 patients diagnosed with temporal lobe epilepsy	Bilateral independent interictal epileptiform activities could be detected in the progress of TLE.

### Dilemmas in Treating Secondary Epileptogenesis

Over the last 30 years, about 30 ASDs have been approved and used to help control epileptic seizures (57). In many conditions, the seizure frequency could be reduced after taking ASDs. However, there are still a certain proportion of patients who would become pharmacoresistant. Compared with patients who had only one seizure focus, those with a secondary focus are more susceptible to pharmacoresistance (58). For pharmacoresistant epilepsy, uncontrolled seizures increase the risk of sudden unexpected death in epilepsy and seriously affect the quality of patients' daily life. Resection of the epileptogenic zones turns to be the optimal option. However, a secondary focus could restrict the surgery's efficiency because the presence of a secondary focus can generate epileptic discharges independently, even when the primary focus is resected.

Some researchers propose that the presence of secondary epileptogenic focus might not account for surgical failure in patients with epilepsy. Take the tumor patients, for instance. Resection of the tumor itself was mostly sufficient for seizure control, even if the mirror focus was spared in the surgery (59). Meanwhile, Goldensohn insisted that EEG evidence of multifocal discharge should not be considered when making decisions regarding epilepsy surgery because follow-up research showed that patients with bilateral foci still have a good prognosis after resection of the unilateral seizure focus (60). A study with 22 patients with temporal lobe neoplasms demonstrated that the mirror focus is not a contraindication for epilepsy surgery. Resection of the primary focus resulted in the disappearance of the secondary focus (51). In contrast, another study argued that the seizure relapses after resection were usually due to secondary foci in homotopic regions contralateral to the primary focus (48). Different phases of secondary epileptogenesis (dependent, intermediate, and independent stages) proposed by Morrell may explain these conflicting reports (13, 47). During the dependent stage, the discharges that originated from the secondary focus are always time-locked to that of the primary focus, which means epileptic discharges at the secondary focus may only be propagated from the primary focus. In the intermediate stage, the discharges of the secondary focus can be different in time phases from that of the primary focus. However, surgical resection of the primary focus can lead to the vanishment of the secondary focus, which means the maintenance of the seizures in the secondary focus needs the existence of the primary focus. Eventually, the secondary focus becomes permanent in the last independent stage. In this stage, the seizure may originate from the secondary focus after the resection of the primary focus. Nevertheless, it should be noted that if the resection of a seizure focus leads to a certain period of seizure-free at first, but followed by a relapse of seizures, other epileptogenic factors such as abnormal stem cells attributed by developmental malformations or tumors may be also taken into account besides secondary epileptogenesis (61).

Thus, although contradictions exist, a conclusion could be drawn that secondary epileptogenesis is one of the major causes of the less favorable surgical outcome. However, the outcome of epilepsy surgery cannot be solely determined on the presence of the secondary focus but should take the different phases into account.

### Possible Mechanisms of Secondary Epileptogenesis

Unlike those neurological diseases with clear pathogenesis, multiple epileptogenic factors include tumor, trauma, neuroinflammation, genetic predisposition, etc., have been shown to result in abnormal excitation in the brain and consequently provoked seizures (1). Moreover, uncontrolled repetitive seizures further aggravate epileptic conditions like secondary epileptogenesis. Given that the genesis of secondary epileptic focus largely depends on the seizure propagation from the primary focus, mechanisms of excitability spreading such as neurotransmission and synaptic plasticity are closely related to secondary epileptogenesis. In the following sections, we discuss the possible mechanisms of secondary epileptogenesis systematically.

## Molecular Mechanisms

### Involvement of Excitotary Neurotransmission

It is widely accepted that the actions of excitatory neurotransmitters play a vital role in the process of seizure propagation. The glutamate-mediated amplified excitatory activity could lead to the recruitment of excitatory neurons and initiation of the hyperactivity, then the spread of the hyperactivity would cause the seizures (62). Pathological excitatory neurotransmission mediated by glutamate receptors has long been regarded as a major factor in clinical and experimental epilepsy etiology. Ionic glutamate receptors include N-methyl-D-aspartic acid (NMDA) receptor, KA receptor, and α-amino-3-hydroxy-5-methyl-4-isoxazole-propionic acid (AMPA) receptor, which conjugate with ion channels to mediate fast signal transduction. Khalilov et al. preliminarily revealed the correlation between ionic glutamate receptors and secondary epileptogenesis in the three-chamber model. They reported that the application of NMDA receptor antagonist on the contralateral hippocampus could prevent the formation of the secondary focus but not the propagation of seizure activity (21), which means NMDA receptors were involved in secondary epileptogenesis caused by the long-lasting synaptic excitatory effects originating from the primary focus. Given that the NMDA receptors were composed of two GluN1 obligatory subunits and two regulatory subunits (63), just as Acutain et al. reported, the decreased expression of GluN2A would further lead to increased seizure susceptibility (64). Thus it can be speculated that changes of the NMDA subunits may also underly secondary epileptogenesis. These findings suggest that the activation of the NMDA receptor is necessary for forming the secondary focus.

Similar to the NMDA receptor, other studies verified the role of the AMPA receptor in secondary epileptogenesis. Barna et al. proposed that intracerebral injection of the AMPA receptor antagonist GYKI-52466 into both the primary and mirror focus led to anticonvulsant effects in anesthetized rats treated by 4-AP (32). Also, the role of the AMPA receptors in secondary epileptogenesis of a KA treated rat model was examined. Interestingly, the application of the AMPA receptor antagonist CNQX led to a priority of seizure generation in the ipsilateral hippocampus, while in the selective KA receptor antagonist and the control group, epileptic discharge mainly originated from the contralateral side (40). Another study further verified this by showing that the AMPA receptor antagonist could reversibly suppress the seizure activity originating from the secondary focus (28). Besides, although barbiturate anesthetics have been widely accepted as a GABAergic function enhancer (65), it is also reported that phenobarbital could modulate the expression of AMPA-type glutamate receptor channels (66). Based on this, Nardou et al. compared the effects of diazepam, unrelated to the AMPA receptors and phenobarbital, on the formation of the mirror focus. They reported that phenobarbital but not diazepam could reduce the occurrence of epileptic spikes in the mirror focus with the presence of GABA and NMDA receptor antagonists (37), which laterally provide evidence for the involvement of the AMPA receptors in secondary epileptogenesis.

In brief, epileptic discharges resulted from neuronal hyperexcitability. The repetitive abnormal epileptic discharges in the primary focus can lead to an increased glutamatergic driving force acting on both excitatory NMDA and AMPA receptors of the possible secondary epileptic focus. This process will decrease the threshold of seizure generation and thus contribute to secondary epileptogenesis.

### The Role of Inhibitory Neurotransmission

Besides excitatory glutamatergic synaptic transmission, inhibitory GABA synaptic transmission also plays a crucial role in maintaining the balance of excitation and inhibition in the central nervous system. Abnormalities in the GABAergic systems lead to declined inhibitory function in the brain, resulting in the dominance of excitation.

Federico et al. examined the role of GABA and glutamate receptor subtypes in the spread of epileptic activity. They confirmed that GABA_A_ but not GABA_B_ receptors were essential in seizure propagation (28). Perfusion of GABA_A_ receptor antagonist bicuculline could lead to spontaneous seizure activity in the isolated guinea pig brain (29). The role of GABA receptors was further verified in a clinical study. Bortolato et al. reported a patient with neural damage in the right frontal lobe and a mirror focus in the contralateral hemisphere. After the resection of the primary focus, the density of GABA_A_/benzodiazepine receptor binding in the left frontal lobe significantly increased (56). A recent study has further reported the role of inhibition defects in the formation of a secondary epileptic focus. The application of bicuculline enhanced contiguous seizure propagation and focal bicuculline microinjection into the regions distant to the 4-AP injection site leading to a secondary, non-synchronous epileptic discharge (67).

Additionally, excitatory GABA action induced by high chloride concentration contributed to seizure generation (68–70). After experiencing recurrent seizures, GABA transmitters directly depolarized neurons due to a persistent increase of extracellular chloride ions termed as a shift of GABA function from inhibitory to excitatory. This early elevated concentration of chloride ions is due to two chloride co-transporters: NKCC1, which imports the chloride ions, and KCC2, which extrudes them. In epileptic conditions, the changed expression of KCC2 and NKCC1 would occasionally influence the ionic homeostasis of chloride ions and contributes to secondary epileptogenesis (71, 72). For example, the GABA-acting ASD phenobarbital is a first-line drug to treat neonatal seizures. However, it would be less efficient after recurrent seizures because phenobarbital exacerbated the high intracellular chloride mediated by a combined action of NKCC1 and the downregulation of KCC2 in an established mirror focus (38). Moreover, although the NKCC1 antagonist bumetanide could not prevent the seizure propagation to the contralateral hippocampus and the formation of the mirror focus, it could block the spontaneous epileptiform activities and partly reduce the excitatory action of GABA in the isolated mirror focus (39). In conclusion, these results suggest the excitatory action mediated by chloride co-transporters can cause a longlasting shift in the depolarizing direction of the actions of GABA and ultimately induce secondary epileptogenesis.

**Figure 1:**
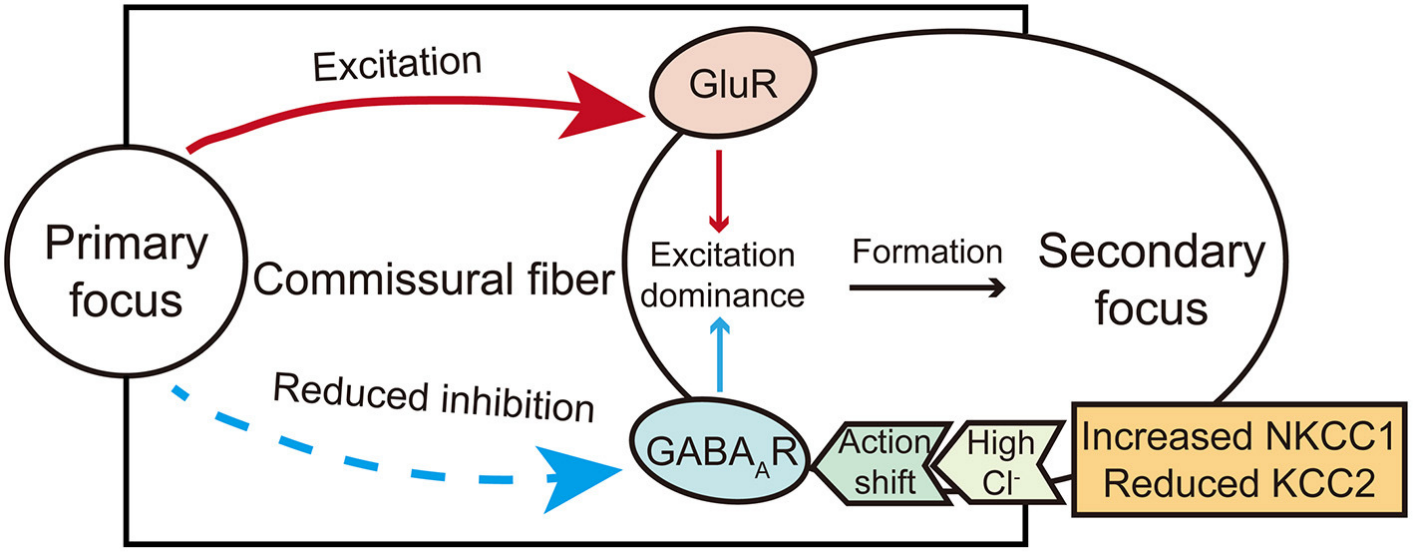
A brief diagram illustrates the action of glutamate and GABA receptors in secondary epileptogenesis. The shift action of GABA from inhibitory to excitatory mediated by chloride co-transporters and excitation actions carried by glutamate commissural fibers co-mediate the formation of the secondary focus.

The formation of the secondary focus is different from that of the primary one and is mainly dependent on synaptic transmission. According to the currently available evidence, the excitatory transmission may mediate the early stage of the secondary focus (dependent and intermediate phase proposed by Morrell). In contrast, the abnormality of inhibitory transmission may further mediate the consolidation stage of the secondary focus (the independent phase). Therefore, the role of these two kinds of synaptic transmission cannot be completely dissected (Figure 1), and further integrating studies are needed to dissect the mechanism of secondary epileptogenesis contributed by both excitatory and inhibitory synaptic transmission.

### Other Molecules Participate in Secondary Epileptogenesis

In the central nervous system, besides the excitatory/inhibitory neurotransmission, which is also the main target of available ASDs, neurons directly connect the cytoplasms of adjacent cells through channels docking of two hemichannels called gap junction (73). It is a common direct pathway for intercellular communication between glial cells and neurons. Connexin 36 (Cx36) mediated gap junction communication had been certified to participate in epileptogenesis and emerge as a potential target for epilepsy (74). Gajda et al. investigated the role of Cx36 mediated gap junction communication in the maintenance and propagation of epileptic discharges in both the primary focus and the mirror focus. They reported that the Cx36 channels also promoted secondary epileptogenesis (35). The above function partially mediated the formation of the mirror focus. They also demonstrated that the level of Cx36 mRNA was significantly increased after experiencing 25–30 spontaneous seizures (33).

On the other hand, cumulative studies demonstrated that epileptic pathogenesis might be associated with other factors which indirectly regulate neural excitability. Such as neurodegeneration, neurogenesis, and neuroinflammation, of which neuroinflammation gradually attracts researchers' attention (75–77). The inflammatory mediators play a significant role in the development of chronic spontaneous seizures (41). Early experiencing febrile seizures can induce the formation of secondary adult epileptogenesis in some cases, which might be mediated by neuroinflammation (78, 79). As Choi et al. reported, the level of HMGB1 showed a significant increase in the serum of patients with febrile seizures at first (80). It significantly contributed to the pathogenesis of hyperthermia-induced seizures and the following epileptogenesis. The application of HMGB1 further aggravated the acquired epilepsy after experiencing febrile seizures, which suggested that it played a vital role in acquired secondary epileptogenesis (81). One of the possible mechanisms may be that HMGB1 induces the expression of P-glycoprotein (P-gp) (82), which is directly related to drug resistance and epileptogenesis (83, 84).

In addition to neuroinflammatory cytokines, other molecules have also been reported to be involved in secondary epileptogenesis. Sulfated octapeptide of cholecystokinin (CCK-8) was a kind of neuropeptide that could increase the firing frequency of the action potentials in the neurons of the hippocampal CA1 (85). Moreover, the decreased calbindin staining might lead to decreased excitability and firing rate of pyramidal cells (86). The unilateral injection of KA into the dorsal hippocampus induced acute status epilepticus, followed by a latent phase with ipsilateral neuronal degeneration, which could generate epileptic seizures. Arabadzisz et al. investigated the expression and distribution of some specific neuromodulators in the hippocampus contralateral to the injection site in this process. The labelings of CCK-8 and calbindin were selectively decreased in the latent phase (34). The authors suggest that such changes in CCK-8 and calbindin expression may relate to the formation of epileptic seizures in the contralateral hippocampus.

Decades of studies have already revealed changes of some crucial molecules in secondary epileptogenesis. Nevertheless, the brain works as an interconnected network. The microscopic molecular mechanisms may not reflect the whole dynamic processes in secondary epileptogenesis. Macroscopical perspectives should be taken into account in different experimental designs.

## Cellular Mechanisms

### Synaptic Plasticity in Secondary Epileptogenesis

In addition to molecular mechanisms, changes in synaptic function were also related to secondary epileptogenesis. The concept “seizures beget seizures” has been widely known for years. Moreover, cumulative studies have proved that repetitive seizures lead to more severe chronic epilepsy (87). In that process, synaptic plasticity certainly plays a key role. The long-term potentiation and depotentiation (LTP and LTD) of synaptic transmission are forms of long-lasting synaptic plasticity in the mammalian brain. During the process of LTP, synaptic strength gradually increases with the repetitive excitatory stimulation, while the situation is inverse in LTD. Both LTP and LTD mediate diverse forms of experience-dependent plasticity, including learning and memory, emotional feelings, and epilepsy (88). The phenomenon of LTP was first found in dentate granule excitatory neurons, which was essential in the stabilization and elimination of synapses during the development and adjustment of neural circuits (89). It is reasonable that the genesis of the secondary epileptic focus is associated with LTP due to that the process of these two phenomena is very similar-both of the two processes require repeated stimulation and reinforcement (90). An electrophysiological study performed by Beldhuis et al. analyzed bilateral hemispheres epileptiform activities on the amygdala kindling rats. The analysis of the linear and non-linear association functions showed that the connection between the two amygdalas was strengthened after daily kindling, and the excitability of the contralateral amygdala increased along with the kindling process (25). Further immunohistochemical studies illustrated that the activation of the neocortical areas contralateral to the primary focus was the result of synaptic connections, repeatedly strengthened synapses could lead to the spread of seizures (31, 55).

Given that the changes in synaptic plasticity are closely related to drug addiction (91). Kirkby et al. provided direct evidence about the role of enhanced synaptic plasticity caused by drug addiction in the secondary kindling process. They used amphetamine as a pretreatment agent to induce addiction in rats, thereby enhancing synaptic plasticity in the brain. Then the relationship between amphetamine pretreatment and the rate of kindling acquisition was studied. They demonstrated that the amphetamine pretreatment would lead to a much faster procedure of the kindling process in the secondary but not the primary epileptic focus (24).

Synaptic plasticity changes eventually lead to the remodeling of neural circuits and can be considered the connection from macroscopic brain network to microscopic synaptic function and transmitters, which provides another perspective for studying secondary epileptogenesis.

### Other Cellular Changes

Other alternative mechanisms for secondary epileptogenesis which were occasionally reported include selective loss of interneurons, formation of excitatory synapses, etc. (87). Also, both mossy fibers sprouting and astrogliosis are reported as biomarkers of aberrant excitatory synaptogenesis, and they were observed in the unilateral KA model and electrical stimulation model (92). Meanwhile, postepileptic lesions showed changes in neuronal density, reactive astrogliosis, and sclerosis of critical structures that might cause secondary epileptogenesis. A histopathological study showed that most patients with TLE would have the characteristics of severe neuronal loss in the amygdala (93). However, the relatively small sample size of these studies limits the utility of these findings, and further systematic studies are required.

### Circuitry Views for Secondary Epileptogenesis

With significant developments in neuroscientific experimental techniques such as optogenetics, trans-synaptic viral tracing, and large-scale single-unit recordings, epilepsy is gradually considered a circuitry disease caused by the formation of abnormal brain networks (94, 95). The study of the time sequence of seizure initiation had demonstrated that the seizures were not just synchronized events in a single region but involved many crucial circuits originating from seizure focus to its downstream regions. The formation of abnormal excitatory circuits usually leads to the generation of seizures. For example, our previous studies have demonstrated that the microcircuit in the subiculum gates the genesis of the generalized seizures and further pharmacoresistance in TLE (68, 96, 97). It can be deduced that the heterogeneity of seizure spread sequence and excitation amplitudes may further lead to the formation of the secondary focus.

After reviewing decades of researches on secondary epileptogenesis, a conclusion can be drawn that the secondary focus was most likely to occur at the homotopic area of the contralateral hemisphere. Because the two hemispheres of the brain, especially in the temporal lobe regions, have the densest neural projection. Our previous study demonstrated that the unilateral kindled amygdala could promote the process of kindling acquisition in the contralateral amygdala (42). Also, it has long been deemed that the secondary epileptic focus can occur not only in the contralateral homotopic areas but also in the other regions of the ipsilateral hemisphere. The independent epileptic discharge could be detected in both the amygdala and the globus pallidus in an epileptic model whose seizures originate from the hippocampus (98). In unilateral amygdaloid overkindled cats, seizures could originate from both the amygdala and the ipsilateral frontal cortex (26). These studies suggest that the secondary epileptic foci do not form in isolation. Generalization of epileptic excitability is usually accompanied by the evolution of epileptic circuits.

Neuroimaging is a valuable tool to visualize the microstructural changes of epileptic circuits. Moreover, isotopic indicators in positron emission tomography (PET) have become one of the most commonly used methods to estimate glucose utilization of a particular nucleus. Handforth et al. compared the behavioral severity with autoradiography anatomic patterns in amygdala-induced status epilepticus. The spread of seizure activities from the amygdala to other limbic and non-limbic structures existed long before the appearance of motor seizures. This network first recruited the direct amygdala projection areas, then the contralateral structures (99). As Bankstahl et al. reported, by applying a novel PET protocol targeting the overactivity of P-gp, seizure-induced regional changes in P-gp activity can be identified (100). The overexpression of P-gp is closely related to epileptogenesis, by which method, the potential secondary epileptic focus can be detected. Diffusion tensor imaging (DTI) changes in patients with TLE were evaluated, and evidence for the microstructural changes of the hippocampus was also provided. Less-robust abnormalities of DTI suggested the secondary involvement of the thalamus in epilepsy. This structure was recruited into the hippocampal epileptic network (54). Additionally, Pustina et al. compared the role of three interhemispheric white matter pathways in generating contralateral epileptiform spikes during interictal activity. Diffusion tensor imaging was used to measure the integrity of those pathways in the temporal lobes: the tapetum, the anterior commissure, and the body of the fornix. These data suggested that the tapetum pathway could cause the emergence of contralateral spikes, and it was not due to the containment of callosal fibers (101).

**Figure 2:**
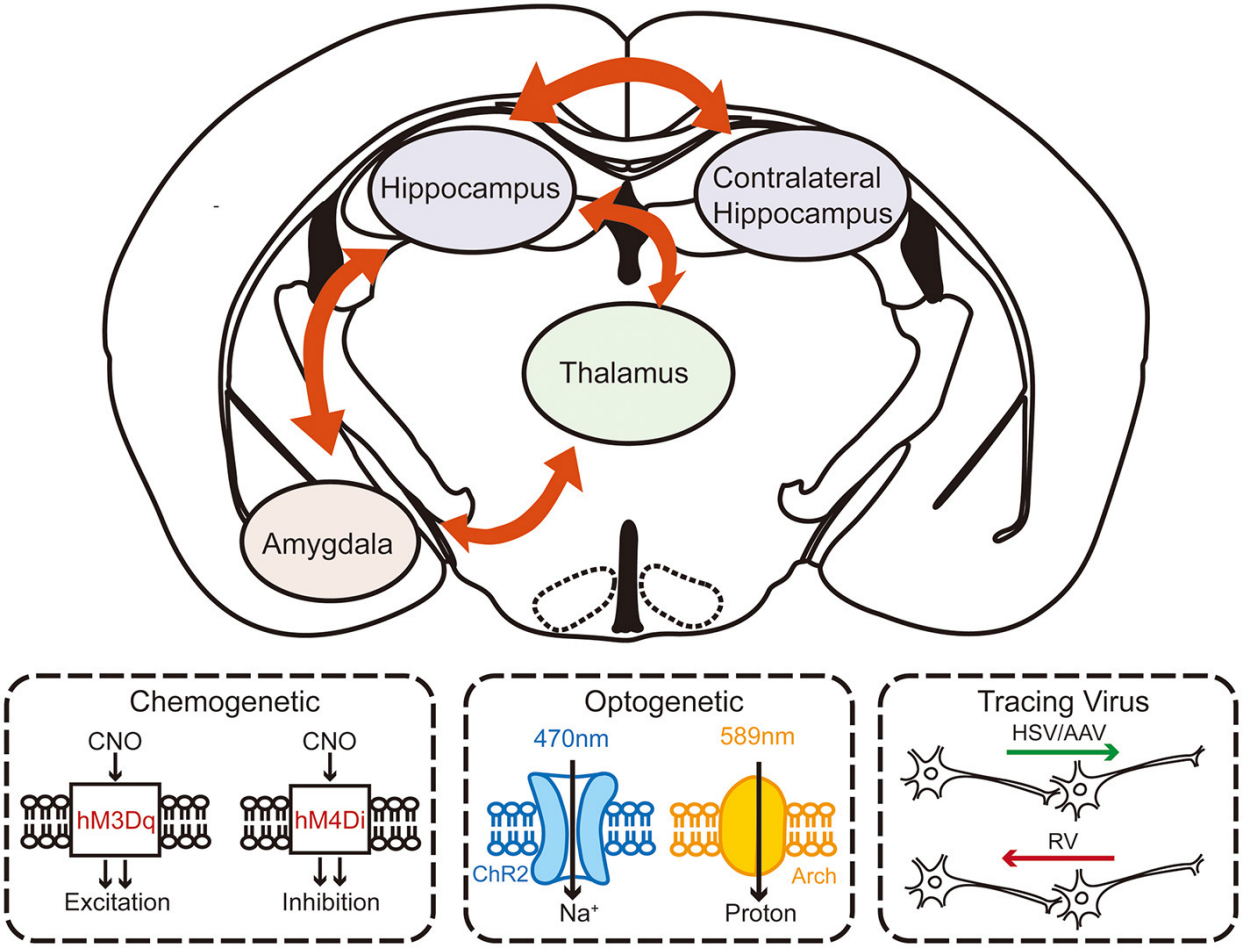
The schematic diagram of investigating the circuity mechanism of secondary epileptogenesis in temporal lobe epilepsy by using advanced experimental approaches. Combined multifaceted techniques including chemogenetics, optogenetics, and viral tracing can help to reveal the possible circuitry basis of secondary epileptogenesis.

Growing evidence suggests that understanding the mechanisms in neurological functions and diseases, especially in epilepsy, cannot be focused solely on the microscopic molecular level. Macroscopic circuitry views provide a more accurate and dynamic mechanism perspective. Although a few studies have already suggested that the formation of the secondary focus is related to the circuits, systematic studies are still needed to elucidate the circuitry mechanism of secondary epileptogenesis (Figure 2).

## Available and Potential Treatments for Secondary Epileptogenesis

### Epilepsy Surgery

In the early 1960s, the development of neurosurgical intervention was just getting started. At that period, ASDs and surgical resection were the main available treatments for epilepsy. For patients with bilateral epileptic foci, neither medication nor surgery can achieve a satisfactory curative effect. However, given that corpus callosum was reported to play a critical role in bilateralization and symmetrization of seizures (30). Some clinicians would choose corpus callosotomy for patients with intractable epilepsy. The curative effect of corpus callosotomy on secondary epileptogenesis was initially demonstrated in animal models. The effect of corpus callosotomy was firstly demonstrated in a motor cortical kindling model. Kudo et al. divided twelve cats into two groups, five with the corpus callosotomy. The corpus callosotomy could significantly delay the seizure progression from focal to generalized convulsive seizures and decline the transfer effect of epileptic seizures (30). Callosotomy was mainly chosen for those patients with complex focal seizures or Lennox-Gastaut syndrome (102). Ono et al. reported that 63.2% of the patients with bilateral discharges showed desirable outcomes after callosotomy for intractable epilepsy. While after correlating postoperative outcomes with EEG data, it turned out that the patients with lateralized seizure discharges often had superior effects compared with those who had bilateral discharges (103).

However, besides the therapeutic effects, side effects should not be ignored. The split brain syndrome as a side effect of callosotomy had been reported due to brain asymmetry. Dyssynchrony of consciousness in the two hemispheres can be observed and further affects the self-care abilities of patients. Sometimes, callosotomy can even ameliorate both generalized seizure and status epilepticus (102). Thus, the callosotomy must be performed with special caution and regarded as the last choice when no alternative options exist.

### Pharmacotherapy

#### Available ASDs

Most of the first and second generation ASDs like phenytoin, valproic acid, and benzodiazepines are used to control seizures in patients with or without secondary focus. Those ASDs act on diverse molecular targets, including voltage-gated sodium/calcium channels and GABA_A_ receptors. Taken that almost no available ASDs can interfere the epileptogenesis (3), the ineffectiveness of these ASDs on secondary epileptogenesis is imaginable.

However, some third-generation drugs act differently. Levetiracetam (LEV) is a pyrrolidone derivative which can be used as both anticonvulsant and antiepileptogenic medication. It does not target postsynaptic receptors or membrane ion channels but acts by combining with the component factor of the synaptic vesicle (SV2A) and further blocks the transmission of excitatory neurotransmission (104). The antiepileptogenic effect of LEV was firstly demonstrated on animal models and can persist after drug withdrawal (105). Yang et al. demonstrated that early administration of LEV could prevent posttraumatic epileptogenesis both *in vivo* and *in vitro*. It also significantly raised the stimulus intensity required to trigger epileptiform bursts (106). Furthermore, combined use of LEV and topiramate could also significantly retard the epileptogenesis in rats after pilocarpine-induced status epilepticus (107). Although direct evidence was still lacking, it can be deduced that LEV might also interfere with the genesis of secondary epileptogenesis.

To sum up, the complete abolishment of secondary epileptogenesis by current ASDs is still not evident. Available ASDs, which mainly target ion channels and GABA receptors, turn out to be invalid for epileptogenesis. Future drug designs should focus on molecules and mechanisms closely related to secondary epileptogenesis.

#### Future Potential Medications

Novel mechanisms findings of secondary epileptogenesis would, in turn, provide potential therapeutic targets for it. For example, both preclinical and clinical evidence has highlighted the importance of neural inflammation on epileptogenesis in recent years. There is positive feedback between the pro-inflammatory factors and the epileptic activities. The biosynthesis of inflammatory cytokines and prostaglandins will be activated after epileptic stimuli and, in turn, enhance the epileptic excitability (108). Thus, the inflammatory inhibitors may have potential antiepileptic effects. We previously reported that targeting the caspase-1-interleukin-1β inflammatory pathway could reduce neuronal excitability and suppress secondary epileptic susceptibility caused by febrile seizures (79, 109). Besides, as mentioned above, the inhibitor of HMGB1 may also have the potential to treat secondary epileptogenesis (81). Additionally, the phenomenon of LTP is based on the function of AMPA receptors and NMDA receptors. (88). Consequently, the AMPA and NMDA receptor antagonist application delays the enhancement of synaptic connection and may prevent the formation of the secondary focus.

Aside from approaches targeted on the molecules closely related to secondary epileptogenesis, directly retarding the primary epileptogenesis may also be helpful (110). New directions of ASDs development should address both the primary and secondary epileptogenesis rather than merely seizure control. With the development of pharmacogenomics and the discovery of accurate biomarkers, precise individualized therapy for secondary epileptogenesis will be possible and could certainly help those patients.

#### Other Treatment Options

**Figure 3:**
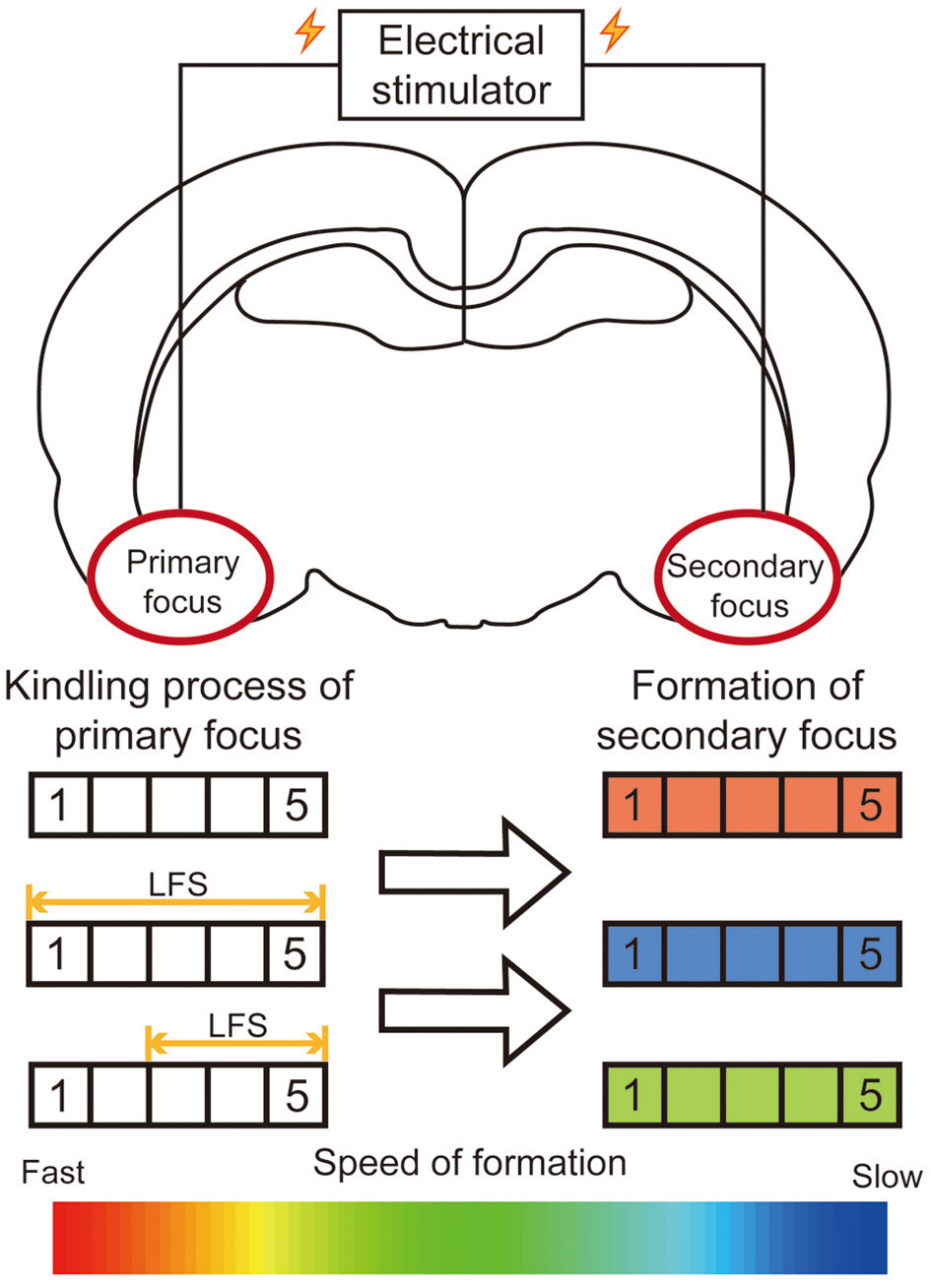
Summary diagram of efficacy of LFS on the primary focus for secondary epileptogenesis. In the kindling model of secondary epileptogenesis, bilateral amygdalae were kindled successively. The application of LFS at the primary focus significantly retards the epileptogenesis of the secondary focus. The rectangle on the left represents the stage to which the primary focus was kindled. The color of the rectangle on the right represents the relative speed of the formation of the secondary focus.

Neurostimulations, including deep brain stimulation, vagus nerve stimulation, and transcranial magnetic stimulation, are effective for neurological diseases (1). Among these, deep brain stimulation has gradually evolved as an effective alternative treatment in epilepsy, with the advantages of reversibility and controllability. For secondary epileptogenesis, our previous research firstly reported that low frequency stimulation (LFS, 1 Hz) at the primary focus could significantly retard the secondary kindling acquisition of the mirror focus (Figure 3). Then we further specified the time window of LFS for secondary epileptogenesis treatment. The LFS would have a better effect before developing into a generalized seizure (42). Similarly, Couturier et al. determined the relative efficacy of different protocols of brain stimulation for secondary epileptogenesis. By comparing the antiepileptic effects of LFS on the corpus callosum and high frequency stimulation (HFS) at both primary focus and anterior nucleus, a conclusion can be drawn that the LFS at the corpus callosum can significantly reduce the seizure frequency of both primary and secondary focus (111). These results provided direct evidence to confirm the promising therapeutic effect of LFS for secondary epileptogenesis.

Recently, the brain-responsive neurostimulator (RNS System, NeuroPace Inc.) has been approved by the FDA as an adjunct treatment for refractory epilepsy, including patients who had more than one epileptic foci (112). In a case report, by implanting an RNS System into a patient whose left temporal seizure focus overlapped with language areas which led to the residual of epileptic structures after surgery, Geller et al. reported that this adjunct treatment achieved a desirable curative effect in that patient (113). Transcranial focal stimulation (TFS), a noninvasive neuromodulation strategy, has been shown to reduce seizure activities and avoid P-gp overexpression in different experimental models. According to these, it is indicated that TFS may also represent a new neuromodulatory strategy to revert secondary epileptogenesis (114).

Despite the promising results, neuromodulation is limited by its invasive nature (associated with device implantation) and battery-related problems. Future studies should focus on the crucial brain regions involved in secondary epileptogenesis and develop more biocompatible and continuable devices.

## Conclusion and Future Prospectives

To sum up, secondary epileptogenesis is a longlasting issue that remains unsolved in epilepsy. Decades of clinical and experimental evidence have confirmed its existence and gradually revealed the possible mechanisms ranging from the molecular to the circuitry level. Both excessive activation of excitatory receptors and reduced inhibition of GABA receptors eventually lead to the formation of the secondary epileptic focus. Other molecules such as HMGB1, caspase-1, and CCK-8 may also contribute to this process. Currently, surgery seems to be the optimal option for secondary epileptogenesis. However, both neuroinflammation inhibitors and DBS show great potential in retarding secondary epileptogenesis. More importantly, with the development of optogenetics and chemogenetics, treatments targeting crucial circuits show great potential in interfering with secondary epileptogenesis. The combination of new neurobiological techniques can bring new insights to illustrate the mechanism of this longlasting problem and novel therapeutic approaches as well.

## Author Contributions

YS wrote the manuscript. YG, YR, CX, and ZC edited the manuscript. All authors contributed to the article and approved the submitted version.

## Funding

This work was supported by grants from the National Natural Science Foundation of China (81630098) and the Research Project of Zhejiang Chinese Medical University (2021JKZKTS010A).

## Conflict of Interest

The authors declare that the research was conducted in the absence of any commercial or financial relationships that could be construed as a potential conflict of interest. The reviewer SW declared a shared affiliation with the authors to the handling editor at the time of review.

## Publisher's Note

All claims expressed in this article are solely those of the authors and do not necessarily represent those of their affiliated organizations, or those of the publisher, the editors and the reviewers. Any product that may be evaluated in this article, or claim that may be made by its manufacturer, is not guaranteed or endorsed by the publisher.
